# An Integrated Meta-Analysis of Two Variants in *HOXA1*/*HOXB1* and Their Effect on the Risk of Autism Spectrum Disorders

**DOI:** 10.1371/journal.pone.0025603

**Published:** 2011-09-29

**Authors:** Ran-Ran Song, Li Zou, Rong Zhong, Xia-Wen Zheng, Bei-Bei Zhu, Wei Chen, Li Liu, Xiao-Ping Miao

**Affiliations:** 1 Department of Maternal and Child Health, School of Public Health, Tongji Medical College, Huangzhong University of Science and Technology, Wuhan, China; 2 Department of Epidemiology and Biostatistics and MOE Key Lab of Environment and Health, School of Public Health, Tongji Medical College, Huazhong University of Science and Technology, Wuhan, China; 3 Guangdong Key Lab of Molecular Epidemiology and Department of Epidemiology and Biostatistics, School of Public Health, Guangdong Pharmaceutical University, Guangzhou, China; University of Ottawa, Canada

## Abstract

**Background:**

*HOXA1* and *HOXB1* have been strongly posed as candidate genes for autism spectrum disorders (ASD) given their important role in the development of hindbrain. The A218G (rs10951154) in *HOXA1* and the insertion variant in *HOXB1* (nINS/INS, rs72338773) were of special interest for ASD but with inconclusive results. Thus, we conducted a meta-analysis integrating case-control and transmission/disequilibrium test (TDT) studies to clearly discern the effect of these two variants in ASD.

**Methods and Findings:**

Multiple electronic databases were searched to identify studies assessing the A218G and/or nINS/INS variant in ASD. Data from case-control and TDT studies were analyzed in an allelic model using the Catmap software. A total of 10 and 7 reports were found to be eligible for meta-analyses of A218G and nINS/INS variant, respectively. In overall meta-analysis, the pooled OR for the 218G allele and the INS allele was 0.97 (95% CI = 0.76-1.25, *P*_heterogeneity_ = 0.029) and 1.14 (95% CI = 0.97-1.33, *P*_heterogeneity_ = 0.269), respectively. No significant association was also identified between these two variants and ASD risk in stratified analysis. Further, cumulative meta-analysis in chronologic order showed the inclination toward null-significant association for both variants with continual adding studies. Additionally, although the between-study heterogeneity regarding the A218G is not explained by study design, ethnicity, and sample size, the sensitive analysis indicated the stability of the result.

**Conclusions:**

This meta-analysis suggests the *HOXA1* A218G and *HOXB1* nINS/INS variants may not contribute significantly to ASD risk.

## Introduction

Autism spectrum disorders (ASD) comprises a pervasive group of neurodevelopmental disorders that share common features of impaired social interaction, deviance in language development, and stereotyped behaviors or narrow range of interests [1]. The prevalence rate of ASD is 0.6%–1% in child and adolescent populations, making it one of the most common disorders of development in the world [2], [3]. The etiology of ASD has been debated ever since, but twin and family studies have highlighted the genetic contribution to ASD, with a hereditability as high as 90% [4]. Converging lines of evidence pointed toward altered prenatal neurodevelopment as being crucial to ASD pathogenesis, suggesting a failure of development of at least one of the rhombomeres, from which facial nucleus and superior olive arose [4]. In this regard, one relied on available biological evidence that genes encoding proteins involved in early neural development could thus underlie this disease [4].

*HOXA1* and *HOXB1*, located on chromosomes 7p and 17q, are two paralogous genes in the HOX gene family of homeobox transcription factors and critically involved in the developing hindbrain during neural tube formation [5]. Evidence from autopsy on brain of an idiopathic autism patient has showed the near absence of the facial nucleus and superior olive [6], while the facial nucleus and superior olive required normal *HOXA1* and *HOXB1* function for their proper development. Similar morphologic deficits in brain have been described in mice knockout *Hoxa1* and *Hoxb1* [7]. Moreover, *Hoxa1* and *Hoxb1* are highly expressed during an early period of development overlapping with the window of maximal prenatal sensitivity in rodent model [8]. These evidence strongly posed *HOXA1* and *HOXB1* as potential candidate genes for ASD. Ingram and his collaborators firstly described variants in *HOX* genes for genetic susceptibility of ASD, including an A to G substitution at base 218 (A218G, rs10951154) of *HOXA1* and a 9-base insertion variant (nINS/INS, rs72338773) of *HOXB1* [9]. Of these two candidate variants, the A218G in *HOXA1* changes a histidine (H) to arginine (R) at position 73 (H73R) and disrupted a string of histidine repeats in exon 1, while the 9-base insertion c.82insACAGCGCCC (referred as INS allele) in exon 1 of *HOXB1* introduces into the amino acid sequence the tripeptide histidine-serine-alanine (H-S-A) [10]. Ingram et al. firstly reported significant deviations from Hardy-Weinberg equilibrium for A218G of *HOXA* in 57 autistic probands and 119 unrelated adults as controls, indicating that the G allele carriers might be susceptible to ASD [9]. Interestingly, interaction among *HOXA1*, *HOXB1*, and gender was also found to be associated with increased risk of ASD [9]. After this first report, a string of studies have subsequently detected these two variants of *HOXA1* and *HOXB1* in ASD, but the results were inconsistent. Inversely, Conciatori et al. pointed out the A allele, not the G allele, was associated with ASD risk in both case-control and family-based association analyses [11]. Additionally, some researches failed to replicate the findings by Ingram et al. [10], [12], [13], [14], [15], [16], [17], [18], [19]. The sample size of any individual study tended to be small and thus potentially leaded to imprecise estimates and inconsistent results for these two variants in ASD. Nevertheless, meta-analysis, due to its exponential increase in sample size, may be a powerful tool to clarify the inconsistent findings in genetic association studies by statistical synthesis of data if properly used [20]. Furthermore, the transmission/disequilibrium test (TDT) based on family is particularly advantageous as less confounding caused by population admixture and is of the same importance as case-control study in genetic association analysis [21]. However, it might be a little statistically challenging to synthesis the family-based studies with case-control studies before Kazeem et al. outlined a methodological improvement for achieving integration of these two different types of studies by a fixed-effects approach, using an allele-based mode [22], and then Nicodemus subsequently extended this method to the random-effects model [23]. Therefore, we conducted a meta-analysis of published studies and applied the method described by Kazeem et al. [22] to integrate the results from case-control and TDT studies to provide more precise evaluation for the association of *HOXA1* and *HOXB1* genetic variants with ASD risk.

## Methods

To ensure the rigour of this current meta-analysis, we designed and reported it according to the Preferred Reporting Items for Systematic Reviews and Meta-analyses (PRISMA) [24] statement and the checklist is shown in table S1 (http://www.prisma-statement.org).

### Search strategy and identification of relevant studies

We searched PubMed, EMBASE, and ISI Web of Science databases for published articles up to August 2010, which had investigated at least one of A218G in *HOXA1* and insertion variant in *HOXB1* associated with ASD in case-control study or TDT study. The search strategy was based on combinations of the keywords ‘*HOXA1*, *homeobox A1*, *HOXB1* or *homeobox B1*’, ‘polymorphism or variant’ and ‘autism or autistic disorder’ without language restrictions. To expand the coverage of our searches, we further performed searches in Chinese Biomedical (CBM) database using the above searching strategy. References of the retrieved articles were also scanned. Reviews, comments, and letters were also checked for additional studies.

The inclusion criteria had to be fulfilled: (1) either case-control or TDT study design; (2) data on any or both polymorphisms of A218G in *HOXA1* and insertion variant in *HOXB1*; (3) presentation of data necessary for calculating odds ratios (ORs); (4) clear definition of ASD. Animal studies, reviews, simply commentaries, case reports and unpublished reports were excluded. Study overlapping with other studies should be eliminated, and the report with complete design or larger sample size was finally selected.

### Data extraction

All data were extracted independently by two reviewers (R–R. Song & L. Zou). The following information was extracted from the eligible studies: first author's name, year of publication, ethnicity, design type of study, and diagnostic criteria for ASD. Counts of alleles in case and control group in case-control studies and numbers of transmitted alleles from heterozygous parents to affected offspring in family-based studies were extracted or calculated from published data in the included studies.

### Statistical analysis

Data from case-control studies were summarized in two-by-two tables, and data from TDT studies were summarized in two-by-one tables. ORs and their 95% confidence intervals (CIs) and standard errors (SEs) were calculated for individual study based on the allele data using the method as described by Kazeem et al. [22]. The *χ* ^2^- based Cochran's Q statistic test was employed to test between-study heterogeneity, and heterogeneity was considered significant when *P*<0.1 for Q statistic. Data from the studies were combined by the random-effects model when heterogeneity was significantly present; otherwise, fixed-effects model was applied. For the synthesis of case-control and TDT studies, the method described by Kazeem et al. was used [22]. After obtain the estimate of logarithm of the OR and its associated SE in each case-control or TDT study, the estimate of combined OR and its associated SE can be calculated by a weighted analysis method [25]. The Catmap software implemented this method for the fix-effects model and plus extended this method for the random-effects model of DerSimonian and Laird to conduct case-control and TDT meta-analysis, which can be download from the comprehensive R network http://www.r-project.org [23]. Overall meta-analysis was initially performed. Then stratified analysis, if feasible, was performed according to study design, sample size, and ethnicity separately. Publication bias was assessed by funnel plot and Egger's test [26]. Additionally, sensitivity analysis was also performed to assess the influence of each study on the overall estimate. Cumulative meta-analysis was also conducted via the assortment of studies by publication time. All *P* values are two-tailed with a significant level at 0.05. All statistical analyses were carried out in Catmap software V1.6.

## Results

### Characteristics of included studies

**Figure 1:**
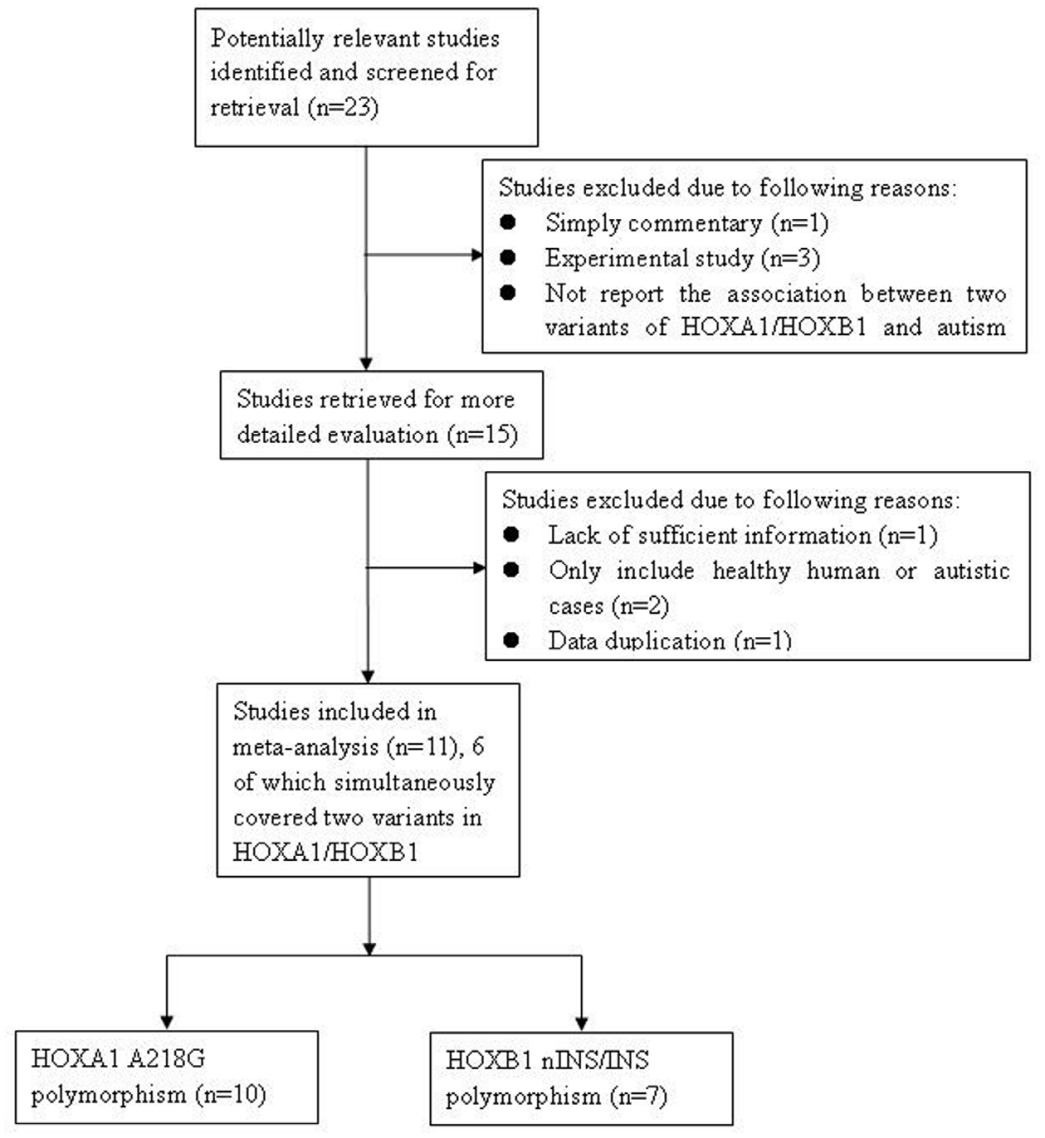
Flow diagram of the study selection procedure.

Figure 1 shows the literature search and study selection procedures. After comprehensive searching, 23 potentially relevant reports were retrieved, of which, 12 reports met the inclusion criteria. However, the study reported by Muscarella et al. in 2007 [16] was excluded since the cases largely overlapped with the sample in analysis by Conciatori et al. [11]. A total of 11 reports were ultimately eligible for this meta-analysis [9], [10], [11], [12], [13], [14], [15], [17], [18], [19], [27]. Additionally, since 5 reports applied a double approach comprising case-control and TDT designs in the same or overlapping probands [9], [11], [15], [19], [27], the case-control studies of these 5 reports were excluded in the overall meta-analysis due to less sample size than TDT studies. Therefore, 10 reports comprising 113 cases, 184 controls and 958 families were relevant to A218G in *HOXA1*, and 7 reports including 113 cases, 184 controls and 570 families were relevant to insertion variant in *HOXB1*. Table 1 shows the characteristics of the included studies.

**Table 1:** Characteristics of included studies.

Study	Publication Year	Ethnicity	Design type	Control matched condition	Case/control (family)	Diagnostic criteria
Ingram [9]	2000	White	Case-control	Ethnicity	57/119	DSM-IV
Ingram [9]	2000	White	Family	–	50	DSM-IV
Li [17]	2002	Mixed	Family	–	110	ADI/ADOS
Devlin [18]	2002	Mixed	Family	–	221	DSM-IV
Talebizadeh [13]	2002	Mixed	Case-control	Ethnicity	35/35	DSM-IV
Collins [19]	2003	White	Case-control	Ethnicity	128/132	DSM IV-R
Collins [19]	2003	Black	Case-control	Ethnicity	64/127	DSM IV-R
Collins [19]	2003	72.7% White; 27.3% Black	Family	–	187	DSM IV-R
Romano [15]	2003	White	Case-control	Ethnicity	*HOXA1*:85/132 *HOXB1*:80/80	DSM-IV
Romano [15]	2003	White	Family	–	85	DSM-IV
Conciatori [11]	2004	White	Case-control	Ethnicity	127/174	DSM-IV
Conciatori [11]	2004	White (Italian)	Family	–	115	DSM-IV
Conciatori [11]	2004	White (American)	Family	–	74	DSM-IV
Gallagher [10]	2004	White	Family	–	78	ADI/ADOS
Yu [12]	2004	Yellow	Family	–	38	ICD-10
Sen [14]	2007	Eastern Indian	Case-control	Ethnicity	62/89	DSM-IV
Sen [14]	2007	North Indian	Case-control	Ethnicity	16/60	DSM-IV
Muscarella [27]	2010	White	Case-control	Ethnicity	169/184	DSM-IV
Muscarella [27]	2010	White	Family	–	247	DSM-IV

### Combining results of case-control and TDT studies

**Figure 2:**
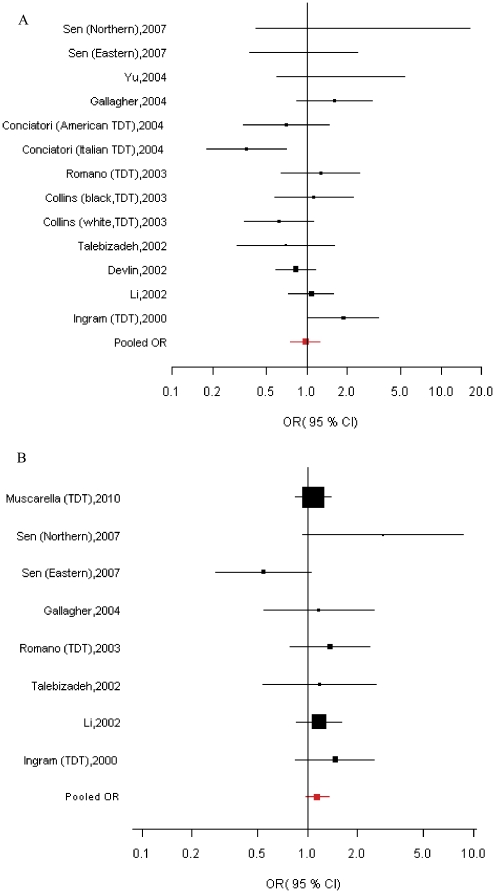
Summary estimates (Odds ratios and 95% confidence intervals) for risk of ASD associated with *HOXA1*/*HOXB1* variants. (A) ASD risk for *HOXA1* A218G: random-effects pooled OR = 0.97, 95%CI = 0.76-1.25, *P* = 0.843; χ^2^ = 22.83, *P*_heterogeneity_ = 0.029. (B) ASD risk for *HOXB1* nINS/INS: fixed-effects pooled OR = 1.14, 95%CI = 0.97-1.33, *P* = 0.118; χ^2^ = 8.78, *P*_heterogeneity_ = 0.269. Abbreviations: CC, case-control study; TDT, transmission/disequilibrium test.

Figure 2A shows the combined result of case-control and TDT studies for A218G in association with ASD. Heterogeneity was found among the 3 case-control and 10 TDT studies from 10 reports (*χ* ^2^ = 22.83, *P*_heterogeneity_ = 0.029); thus, the random-effects model was employed. Pooled OR and 95% CI were calculated for the 218G allele versus the 218A allele. However, no significant association was found between the allelic variant and ASD risk (OR = 0.97, 95%CI = 0.76-1.25, *P* = 0.843). For *HOXB1* nINS/INS variant, no evidence of heterogeneity was presented among the 3 case-control and 5 TDT studies from 7 reports (*χ* ^2^ = 8.78, *P*_heterogeneity_ = 0.269). In the fixed-effects model, this insertion variant was not significantly associated with risk of ASD (OR = 1.36, 95%CI = 0.97-1.33, *P* = 0.118; Figure 2B).

### Stratified analysis

The stratified analysis was firstly performed by study design. In case-control studies, evidence of between-study heterogeneity was found for *HOXA1* variant (*χ* ^2^ = 13.14, *P*_heterogeneity_ = 0.069; Table 2). The pooled allelic OR in the random-effects model was 0.91 (95%CI = 0.67-1.26, *P* = 0.588). There was no indication of heterogeneity for *HOXB1* variant (*χ* ^2^ = 8.83, *P*_heterogeneity_ = 0.116; Table 3). The pooled OR of the INS allele versus nINS allele in the fixed-effects model was 1.13 (95%CI = 0.90-1.42, *P* = 0.284). In TDT studies, the heterogeneity test for A218G showed positive results (*χ* ^2^ = 21.13, *P*_heterogeneity_ = 0.012). In the random-effect model, this variant presented no association with ASD risk (OR = 0.98, 95%CI = 0.74-1.31, *P* = 0.900). No sign of heterogeneity was detected for *HOXB1* insertion variant (*χ* ^2^ = 1.34, *P*_heterogeneity_ = 0.854), and no significant association between ASD and this variant was found in the fixed-effects model (OR = 1.16, 95%CI = 0.98-1.38, *P* = 0.079).

**Table 2:** Meta-analysis of case-control and TDT studies between the A218G in *HOXA1* and ASD.

Study ID	Case-control		TDT		OR(95%CI)	
	Cases	Controls	Transmitted G	Non-transmitted G	Case-control	TDT
	A/G	A/G				
Ingram, 2000 [9]	-	-	30	16	-	1.88 (1.02-3.44)
Ingram, 2000 [9]	91/23	212/26	-	-	2.06 (1.12-3.80)	-
Li, 2002 [17]	-	-	54	50	-	1.08 (0.74-1.59)
Devlin, 2002 [18]	-	-	63	76	-	0.83 (0.59-1.16)
Talebizadeh, 2002 [13]	58/12	54/16	-	-	0.70 (0.30-1.61)	-
Collins (White), 2003 [19]	-	-	18	29	-	0.62 (0.34-1.12)
Collins (Black), 2003 [19]	-	-	18	16	-	1.13 (0.57-2.21)
Collins (White), 2003 [19]	215/41	220/44	-	-	0.95 (0.60-1.52)	-
Collins (Black), 2003 [19]	59/69	110/144	-	-	0.89 (0.58-1.37)	-
Romano, 2003 [15]	-	-	19	15	-	1.27 (0.64-2.49)
Romano, 2003 [15]	150/20	224/40	-	-	0.75 (0.42-1.33)	-
Conciatori (Italian), 2004 [11]	-	-	11	31	-	0.35 (0.18-0.71)
Conciatori (American), 2004 [11]	-	-	12	17	-	0.71 (0.34-1.48)
Conciatori, 2004 [11]	237/17	304/44	-	-	0.50 (0.28-0.89)	-
Gallagher, 2004 [10]	-	-	24	15	-	1.60 (0.84-3.05)
Yu, 2004 [12]	-	-	9	5	-	1.80 (0.60-5.37)
Sen (Eastern Indian), 2007 [14]	116/8	166/12	-	-	0.95 (0.38-2.41)	-
Sen (Northern Indian), 2007 [14]	30/2	117/3	-	-	2.60 (0.42-16.27)	-
Total	956/192	1407/329	258	270	0.92^a^ (0.67-1.26)	0.98^b^ (0.74-1.31)

a Random-effects pooled OR, *P* = 0.588; *χ* ^2^ = 13.14, *P*_heterogeneity_ = 0.069.

b Random-effects pooled OR, *P* = 0.900; *χ* ^2^ = 21.13, *P*_heterogeneity_ = 0.012.

**Table 3:** Meta-analysis of case-control and TDT studies between the insertion variant in *HOXB1* and ASD.

Study ID	Case-control		TDT		OR(95%CI)	
	Cases	Controls	Transmitted INS	Non-transmitted INS	Case-control	TDT
	nINS/INS	nINS/INS				
Ingram, 2000 [9]	-	-	32	22	-	1.45 (0.85-2.50)
Ingram, 2000 [9]	85/29	186/52	-	-	1.22 (0.72-2.06)	-
Li, 2002 [17]	-	-	86	74	-	1.16 (0.85-1.59)
Talebizadeh, 2002 [13]	53/17	55/15	-	-	1.18 (0.53-2.59)	-
Romano, 2003 [15]	-	-	30	22	-	1.36 (0.79-2.36)
Romano, 2003 [15]	126/34	124/36	-	-	0.93 (0.55-1.58)	-
Gallagher, 2004 [10]	-	-	14	12	-	1.17 (0.54-2.52)
Sen (Eastern Indian), 2007 [14]	110/14	144/34	-	-	0.54 (0.28-1.05)	-
Sen (Northern Indian), 2007 [14]	26/6	111/9	-	-	2.85 (0.93-8.70)	-
Muscarella, 2010 [27]	-	-	127	118	-	1.08 (0.84-1.38)
Muscarella, 2010 [27]	262/76	303/65	-	-	1.35 (0.93-1.96)	-
Total	662/176	923/211	289	248	1.13^a^ (0.90-1.42)	1.16^b^ (0.98-1.38)

a Fixed-effects pooled OR, *P* = 0.284; *χ* ^2^ = 8.83, *P*_heterogeneity_ = 0.116.

b Fixed-effects pooled OR, *P* = 0.079; *χ* ^2^ = 1.34, *P*_heterogeneity_ = 0.854.

The data were further stratified by sample size into 2 subgroups, the large-sample-size subgroup (number of cases in case-control study or number of family in family-based study >80) and the small- or moderate-sample-size subgroup (number of cases in case-control study or number of family in family-based study ≤80). Heterogeneity was detected for A218G in the large-sample-size subgroup including 5 TDT studies (*χ* ^2^ = 10.10, *P*_heterogeneity_ = 0.039) but not in the small-sample size subgroup including 3 case-control studies and 5 TDT studies (*χ* ^2^ = 7.90, *P*_heterogeneity_ = 0.342). No significant association between A218G and ASD risk was found in the small- or large-sample size subgroup (OR = 1.22, 95%CI = 0.93-1.62 and OR = 0.83, 95%CI = 0.68-1.03, respectively). For *HOXB1* insertion variant, no significant associations were observed in both the large-sample-size subgroup including 3 TDT studies (OR = 1.14, 95% CI = 0.94-1.36, *P*_heterogeneity_ = 0.733) and the small-sample size subgroup including 3 case-control studies and 2 TDT studies (OR = 1.14, 95% CI = 0.83-1.57, *P*_heterogeneity_ = 0.090).

In terms of ethnicity, the data were stratified into Whites, Blacks, Yellows, and Indians. No statistically significant finding was seen in Whites for A218G or nINS/INS variant. The pooled OR for the former was 0.92 (95% CI = 0.55-1.54, *P*_heterogeneity_ = 0.002) and the latter was 1.17 (95% CI = 0.95-1.47, *P*_heterogeneity_ = 0.719). The pooled OR could not be appraised in Blacks, Yellows, and Indians because of limited numbers of studies conducted in these populations.

### Sensitivity analyses for combined studies of HOXA1 polymorphism

Given the significant between-study heterogeneity for *HOXA1* A218G polymorphism, we conducted a sensitive meta-analysis to assess the effects of each individual study on the combined OR. A random-effect model was employed since heterogeneity was indicated. A series of combined OR with 95% CIs produced repeatedly after removal of each particular study consistently encompassed 1.0, suggesting the stability of the outcome that A218G was not associated with ASD risk (Table 4). Additionally, sensitivity analyses indicated that TDT study conducted in Italian families by Conciatori et al. [11] was the main origin of the heterogeneity for *HOXA1* variant. The *P* value for Q test was not less than 0.1 after deletion of this study by Conciatori et al. (*χ* ^2^ = 19.99, *P*_heterogeneity_ = 0.130), but the effect of 218G still lacked of significance (OR = 1.03, 95% CI = 0.89-1.19, *P* = 0.67).

**Table 4:** Sensitivity analysis of pooled OR combining family-based and case-control studies for *HOXA1* A218G polymorphism.

Study omitted	OR (95%CI)	*P* a	*P* b_heterogeneity_
Ingram (TDT), 2000 [9]	0.91 (0.72-1.16)	0.456	0.088
Li, 2002 [17]	0.96 (0.72-1.29)	0.805	0.022
Devlin, 2002 [18]	1.00 (0.75-1.34)	0.991	0.025
Talebizadeh, 2002 [13]	1.00 (0.77-1.30)	0.984	0.022
Collins (White, TDT), 2003 [19]	1.02 (0.78-1.33)	0.884	0.038
Collins (Black, TDT), 2003 [19]	0.96 (0.73-1.26)	0.794	0.020
Romano (TDT), 2003 [15]	0.95 (0.73-1.25)	0.733	0.023
Conciatori (Italian TDT), 2004 [11]	1.04 (0.84-1.29)	0.698	0.216
Conciatori (American TDT), 2004 [11]	1.00 (0.77-1.31)	0.998	0.023
Gallagher, 2004 [10]	0.93 (0.72-1.20)	0.591	0.042
Yu, 2004 [12]	0.95 (0.73-1.22)	0.693	0.028
Sen (Eastern Indian), 2007 [14]	0.98 (0.75-1.27)	0.867	0.019
Sen (Northern Indian), 2007 [14]	0.96 (0.74-1.23)	0.743	0.027

Abbreviations: TDT, transmission/disequilibrium test.

a DerSimonian and Laird Random-effects model used to determine the significance of the overall OR.

b Cochran's *x* ^2^-based Q statistic test used to assess the heterogeneity.

### Cumulative meta-analyses

**Figure 3:**
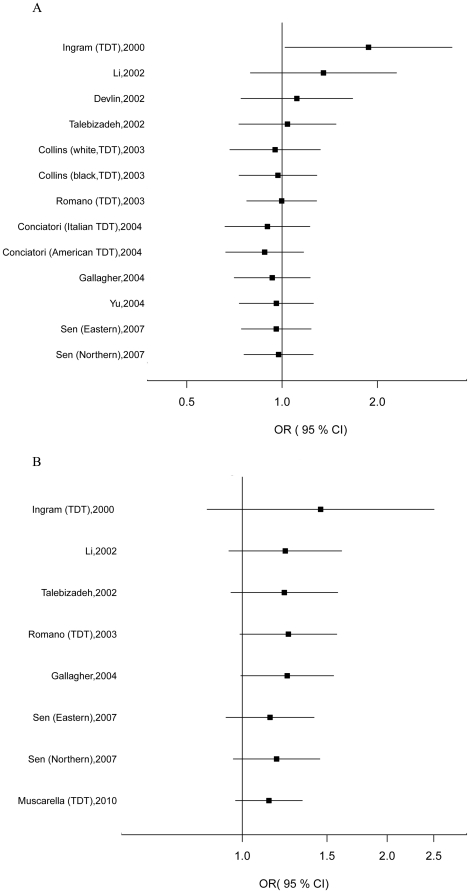
Results of cumulative meta-analysis of association between *HOXA1*/*HOXB1* variants and ASD risk. (A) Cumulative forest plot for A218G using the random-effects model. (B) Cumulative forest plot for nINS/INS using the fixed-effects model.

Cumulative meta-analyses of these two variants were also conducted via assortment of studies in chronologic order. Figure 3A and Figure 3B show the results from the cumulative meta-analyses for the *HOXA1* A218G in the random-effects model and the *HOXB1* nINS/INS in fixed-effects model. The effect of A218G and nINS/INS both tended to be null significant association over time. Moreover, the 95% CIs became increasingly narrower with increasing data, suggesting that the precision of the estimates was progressively enhanced by continual adding more studies.

### Publication bias

As reflected by funnel plots (Figure S1 and Figure S2) and Egger's test, there was no publication bias for A218G and nINS/INS variants (*P* = 0.564 and 0.591, respectively).

## Discussion

This current meta-analysis included 113 cases, 184 controls, and 958 families concerning the A218G in *HOXA1* and 113 cases, 184 controls, and 570 families concerning the insertion variant in *HOXB1*. Both the A218G and nINS/INS variants showed no significant association with risk of ASD. This current meta-analysis, to the best of our knowledge, firstly integrated the case-control and TDT studies to reflect the precision effect of *HOXA1* and *HOXB1* variants in ASD risk.

*HOXA1* is a critical member of HOX gene family involved in the development of hindbrain [5]. Given the critical role in development of brain stem, *HOXA1* was of special interest in ASD. In the *HOXA1* gene, the A218G variant causing a histidine to arginine has been shown to disrupt the string of histidine repeats, which was believed to be the binding site of other proteins [9]. According to the function of this variant, studies continuously measure this variant in ASD risk, but with mixed or conflicting results. In this meta-analysis, neither the overall combined analysis nor the stratified analysis showed association between A218G and ASD with evidence of heterogeneity. To explore the source of heterogeneity, we further conducted stratified and sensitive analyses. Analysis stratified by sample size suggested that the heterogeneity was only presented in the large-sample-size subgroup, while sensitive analysis showed the heterogeneity was effectively removed after deletion of the TDT study in Italian by Conciatori et al. [11]. Interestingly, the large-sample-size subgroup comprised this TDT study by Conciatori et al., indicating this report was the main origin of the heterogeneity. Under review of this report, Conciatori et al. indicated a contradictory result to other included studies that the 218A allele, not the 218G allele, increased risk of ASD. After removing this study, the A218G variant still lacked significance in ASD. Moreover, effect estimates did not change significantly after in turn removing other studies, indicating the stability of this current result. The cumulative analysis gave further support to this current result by showing tendency of A218G toward to null-significant association after 2002 over time. In view of these, we are convinced that the result of the null-significant association between A218G variant and ASD is sound and reliable.

*HOXB1*, in conjunction with *HOXA1*, partly determined the placement of hindbrain segments in the proper location along the anterior-posterior axis during embryo development [5]. A 9-bp insertion variant, located in the amino-terminal coding region and introduces the tripeptide histidine-serine-alanine in the exon 1 of the *HOXB1* gene [28], has been investigated by multiple studies in relation to ASD risk, but results were conflicting [9], [10], [13], [14], [15], [17], [27]. In this study, no significant association of this HOXB1 variant and ASD was identified in overall meta-analysis or stratified analyses by study design, sample size, and ethnicity under fixed-effect model. The cumulative analysis further reported the null-significant association tended to be stable as the number of studies and the sample size increased, conceivably suggesting no association of HOXB1 variant with ASD risk.

Integrating case-control and family based studies is an important aspect of genetic analysis, and combined estimates can provide an overall picture of the effect size attributable to genetic polymorphism. An illustrative application has been made by Gong et al. to *Neuregulin 1* polymorphisms in schizophrenia [29]. In this meta-analysis, when the case-control and family studies were appraised independently, no significant association was found for the *HOXA1* or *HOXB1* variant, indicating the statistical consistency between these two types of studies. When the case-control studies were combined with family studies, statistical power was enhanced from enlargement of sample size, and the ORs and *P* values stably showed the null-significant associations. This meta-analysis integrating case-control with family-based studies therefore provides a straightforward mean to increase the ability to clarify the conflicted results of genetic studies.

Despite the clear strength of this study integrating case-control and family studies, some limitations merit serious consideration. Some heterogeneous natures of studies, including mixed population samples, variant age range of controls in case-control design, multiplex or simplex trios utilized in family studies, possibly have effect on the current result. However, due to lack of detail data or limited small number of some studies, we were unable to perform further analysis. Given the *HOXB1* and *HOXA1* genes have synergized in patterning hindbrain structures, there was a hypothesis that gene-gene interaction between *HOXA1* and *HOXB1* variants might be involved in ASD. However, interaction of these two variants could not be appraisal in this meta-analysis because of a lack of special data.

In conclusion, this meta-analysis helped for strongly clarifying the discrepancies of genetics studies into associations of *HOXA1* and *HOXB1* variants with ASD, and suggest null association of the A218G and nINS/INS variants with ASD risk. Further analysis should be imposed in possible interaction effect between *HOXA1* and *HOXB1* genetic polymorphisms in modulation of ASD risk.

## Supporting Information

Table S1 **PRISMA Checklist for this meta-analysis.**
(DOC)

Figure S1 **The funnel plots of the** ***HOXA1*** **A218G variant for ASD.** Egger's test: *P* = 0.564.
(TIF)

Figure S2 **The funnel plots of the** ***HOXB1*** **nINS/INS variant for ASD.** Egger's test: *P* = 0.591.
(TIF)
